# Apcin inhibits the growth and invasion of glioblastoma cells and improves glioma sensitivity to temozolomide

**DOI:** 10.1080/21655979.2021.2003927

**Published:** 2021-11-30

**Authors:** Yiming Ding, Chuanbao Zhang, Lei He, Xinyu Song, Chengjun Zheng, Yuchu Pan, Shuqing Yu

**Affiliations:** Department of Neurosurgery, Beijing Tiantan Hospital, Capital Medical University, Beijing, China

**Keywords:** Glioblastoma, CDC20, apcin, temozolomide

## Abstract

Glioblastoma (GBM) is the most common malignant primary brain tumor, and GBM patients have a poor overall prognosis. CDC20 expression is increased in a variety of tumors and associated with temozolomide (TMZ) resistance in glioma cells. Apcin specifically binds to CDC20 to inhibit APC/C-CDC20 interaction and exhibits antitumor properties. The purpose of this article was to assess whether apcin inhibits tumor growth in glioma cell lines and increases the sensitivity of GBM to TMZ. In this study, a series of biochemical assays, such as Cell Counting Kit-8 (CCK-8), wound healing, apoptosis and colony formation assays, were performed to determine the antitumor properties of apcin in glioma cells. GBM cell apoptosis was detected by western blotting analysis of related proteins. Apcin increased the sensitivity of glioma to TMZ, as confirmed by CCK-8 and western blotting analysis. The results showed that apcin significantly inhibited the proliferation of glioma cells in a time- and dose-dependent manner. The migration decreased with increasing apcin concentrations. Increased Bim expression indicated that apcin promotes the apoptosis of glioma cells. Furthermore, apcin improved glioma sensitivity to TMZ. The results showed that apcin can effectively inhibit GBM growth and improve TMZ sensitivity. Apcin has the potential to treat GBM and is expected to provide new ideas for individualized treatment.

## Introduction

Glioblastoma (GBM), the most common malignant primary brain tumor, accounts for 57% of all gliomas. Patients with GBM have a poor overall prognosis and a low long-term survival rate of 5.8%, even though they undergo multiple treatments [1]. The main reasons for poor patient prognosis are tumor recurrence and chemotherapy resistance [2]. Temozolomide (TMZ) is the most commonly used first-line chemotherapeutic agent in GBM, and high levels of MGMT promoter methylation can reduce the efficacy of standard TMZ therapy [3]. Therefore, the development of new therapeutic agents for combination with TMZ could improve the survival rate of overall survival patients.

Cell division cycle protein 20 homolog (CDC20), a key factor of the anaphase-promoting complex or cyclosome (APC/C), has an important role in chromosome segregation and mitotic exit [4]. CDC20 plays a key role in apoptosis and promotes tumor development by mediating specific signaling pathways [5]. Recent studies have shown that CDC20 also plays an important role in carcinogenesis and cancer progression and has the potential to be a promising therapeutic target [6]. High expression of CDC20 has been observed in different types of human cancers, such as lung cancer, breast cancer, colorectal cancer, gastric cancer, oral squamous cell carcinoma, pancreatic ductal adenocarcinoma, prostate cancer, and urothelial bladder cancer, and is associated with a poor prognosis [7].

Bim is a member of the BCL-2 family [8]. Bim has been the most widely studied in member of the antiapoptotic protein Bcl-2 family. Bim is a key factor in the induction of apoptosis in tumors, and apoptosis in tumor cells is sensitive to changes in Bim protein expression levels [9]. Drugs mimicking the effects of the BH3-only proteins have been shown to induce apoptosis in different cancer [10]. Therefore Bim can be used as a marker of apoptosis.

Apcin specifically binds to CDC20 to inhibit APC/C-CDC20 interaction, thereby competitively inhibiting APC/C-dependent ubiquitination [11]. Recent studies have shown that APC/C inhibitors are promising therapeutic agents for cancer [12]. The present study found that apcin suppresses metastasis in triple-negative breast cancer and inhibits the growth and invasion of osteosarcoma cells [13,14]. However, the antitumor properties of apcin in GBM have not yet been studied.

The purpose of this article was to assess whether targeted inhibition of CDC20 could improve the sensitivity of GBM to TMZ. Our findings revealed that apcin inhibits tumor growth in glioma cell lines and increases the sensitivity of GBM cells to TMZ.

## Materials and methods

### Cell culture

U251MG cells were provided by the Chinese Academy of Sciences (Shanghai, China). TMZ-resistant U251MG(U251TR) cells were obtained from iCell Bioscience Inc (Shanghai, China). Cells were cultured in Dulbecco’s Modified Eagle’s Medium (DMEM; Gibco; Thermo Fisher Scientific, USA) supplemented with 10% fetal bovine serum (FBS; Gibco; Thermo Fisher Scientific, USA) in a standard humidified incubator under 5% CO^2^ at 37°C.

### Reagents

Apcin (Catalog No. HY-110287) was purchased from MedChemExpress. Cell Counting Kit-8 (CCK-8; Dojindo, Kumamoto, Japan) was used to determine cell viability. Primary antibodies for Bim (1:1,000; cat. no. sc-374,358) were purchased from Santa Cruz Biotechnology, Inc.

### Cell viability assay

Cell viability was assessed by using CCK-8 assays [15]. First, cells were seeded in 96-well plates at a density of 5,000 cells per well. After overnight incubation, the cells were treated with different concentrations of apcin and cultured for 24, 48 and 72 h. At the end of the treatment, 100 μL of DMEM and 10 μL of CCK-8 were added to each well and incubated for 2 h. Finally, the absorbance was measured at 450 nm using an Infinite M200 PRO plate reader (Tecan, Switzerland).

### Cell apoptosis assay

U251MG cells (5x10^5^ cells/well) were seeded in 6-well plates and treated with different concentrations of apcin for 48 h. Subsequently, the cells were harvested in 500 µl of binding buffer with 5 µL of propidium iodide (PI) and 5 µL of annexin V-fluorescein isothiocyanate (FITC) for 15 min at 4 °C in the dark. Apoptosis was analyzed by flow cytometry (Accuri C6; BD Biosciences, USA). The results were analyzed using FlowJo software.

### Wound healing assay

U251MG cells (5x10^5^ cells/ml) were seeded on a 6-well plate and incubated until the cell monolayer grew to almost 80% confluence. The direction of cell migration was determined by creating a straight line wound using a sterile 100 µl pipette tip. After a period of time, the cells migrated to fill the wound area, and microscopic examination was performed. Images were captured at 0 h and 48 h. The images were compared to quantify the migration rate of the cells. The results were analyzed using ImageJ software.

### Western blot assay

Cells were lysed using RIPA buffer (APPLYGEN, Beijing, China) containing a protease inhibitor cocktail. A gel with a suitable concentration (BioSci: 8,012,011) was selected to separate protein (30 μg) from each sample, after which the proteins were transferred to a nitrocellulose membrane. The membranes were blocked with 3% bovine serum albumin (BSA) in Tris-buffered saline with 0.1% Tween 20 (TBST) for 1 h at room temperature, followed by incubation with the primary antibody of the corresponding antigen overnight at 4°C. After incubation, the membrane was washed with TBST 3 times and incubated with fluorescently labeled secondary antibody for 1 h at room temperature. After three washes in TBST, specific protein bands were detected using the Odyssey infrared imaging system (LI-COR, Lincoln, NE) [16]. GAPDH was used as an internal control for sample loading and standardization.

### Colony forming cell assay

Cells (500 cells/well) were seeded on 6-well culture plates and cultured in DMEM supplemented with 10% FBS. The cells were treated with the indicated agents and incubated for 10 days at 37°C and 5% CO_2_. The colonies were then stained with 0.1% crystal violet and counted. For each set of colonies, three independent assays were carried out. The results were analyzed using ImageJ software.

### Statistical analysis

Each experiment was repeated three times. All statistical analyses were conducted using SPSS or GraphPad Prism 7. Differences with *p* < 0.05 were considered statistically significant.

## Results

Recently, emerging evidence suggests that apcin can significantly inhibit osteosarcoma growth and can promote tumor apoptosis. In addition, the problem of TMZ resistance seriously affects the prognosis of glioma patients, and there is an urgent need to develop drugs to improve the sensitivity of glioma to TMZ. Here, we hypothesized that targeted inhibition of CDC20 could improve the sensitivity of GBM to TMZ. We first demonstrated that apcin suppresses glioma cell proliferation, and then our study demonstrated that apcin increases the TMZ sensitivity of glioma cells. to treat GBM and is expected to provide new ideas for individualized treatment.

### Apcin suppresses glioma cell proliferation

**Figure 1.**
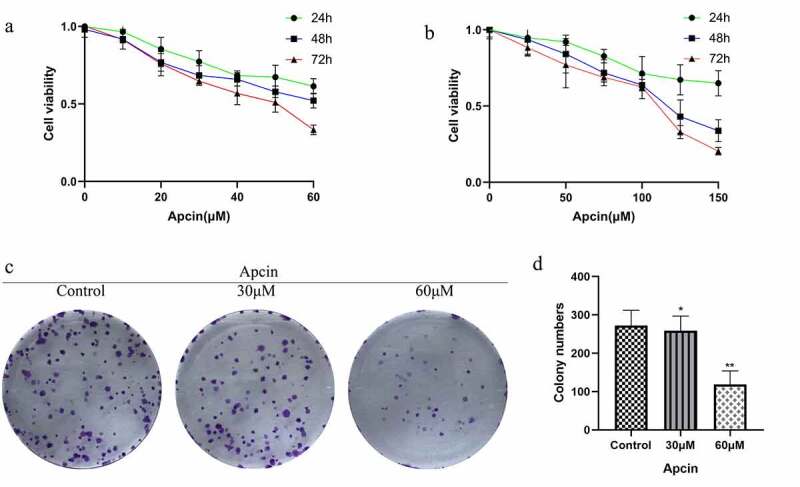
Apcin suppresses glioma cell proliferation. (a) The CCK-8 assay was performed after treating U251MG cells with the indicated concentrations of apcin for 24, 48 and 72 h. (b) The CCK-8 assay was performed after treating U87MG cells with the indicated concentrations of apcin for 24, 48 and 72 h. (c) Colony formation assay showing the sensitizing effects on GBM cells after apcin treatment. (d) Quantitative results of Colony formation assay (*p < 0.05 vs. the control group, **p < 0.01 vs. the control group)

We first assessed whether apcin inhibited the proliferation of human glioma cells. The CCK-8 assay was performed after treating U251MG cells with the indicated concentrations of apcin for 24, 48 and 72 h. The results showed that treatment with 30 μM apcin caused slight GBM cell growth inhibition at 24 h but almost 50% inhibition at 48 h and 72 h. However, treatment with 60 μM apcin at 48 h and 72 h resulted in approximately 50% and approximately 75% inhibition of U251MG cell growth (IC50:30.77 μM) (Figure 1(a)). Similarly, U87MG cell growth was significantly inhibited when the concentration of apcin was greater than 100uM (IC50:81.38 μM) (Figure 1(b)). The results showed that apcin significantly inhibited the proliferation of glioma cells in a time- and dose-dependent manner. The colony formation of U251MG cells after 60 μM apcin treatment was significantly lower than that of the control group, and the colony formation of U251MG cells decreased with increasing apcin concentration (Figure 1(b,c)). These results suggest that apcin shows antitumor properties in glioma cells.

### Apcin induces glioma cell apoptosis

**Figure 2.**
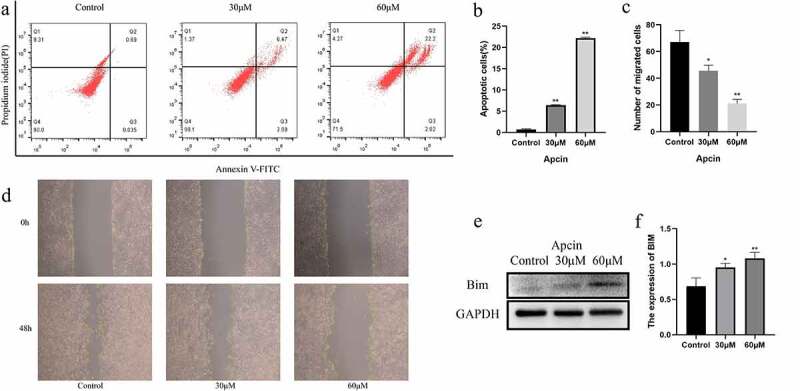
Apcin induces glioma cell apoptosis. (a) U251MG cells were stained with PI and annexin V-FITC for apoptotic analysis after were treated with 0, 30 or 60 μM apcin for 48 h. (b) Quantitative results of colony formation assay. (c, d) The migration ability of GBM cells after apcin treatment was assessed using a wound healing assay. (e, f) The expression of Bim after apcin treatment was detected using western blotting (*p < 0.05 vs. the control group, **p < 0.01 vs. the control group)

We further investigated the relationship between apcin and apoptosis in glioma cells. The CCK-8 assay showed that the effect of apcin on glioma cell viability was already significant after 48 h. Therefore, U251MG cells were treated with the indicated concentrations of apcin for 48 h. Apoptosis was assessed by a PI/FITC-annexin V assay. We found that apcin treatment significantly induced apoptosis in a dose-dependent manner (Figure 2(a,b)). These results suggest that apcin inhibits the proliferation of glioma cells by inducing apoptosis.

### Apcin inhibits glioma cell migration

We determined whether apcin inhibits the migration of U251MG cells by wound healing assay. After a straight line was scratched to create a wound, the cells were incubated with apcin for 48 h. The migration of cells was significantly inhibited after apcin treatment, and the migration decreased with increasing apcin dose (Figure 2(c,d)).

### Apcin induces Bim expression

We performed western blot experiments to further determine the mechanism by which apcin promotes the apoptosis of glioma cells. The results showed that the expression of Bim was increased in U251 cells after apcin treatment (Figure 2(e,f)). Moreover, apcin promoted Bim expression in a dose-dependent manner.

### Apcin increases the TMZ sensitivity of glioma cells

**Figure 3.**
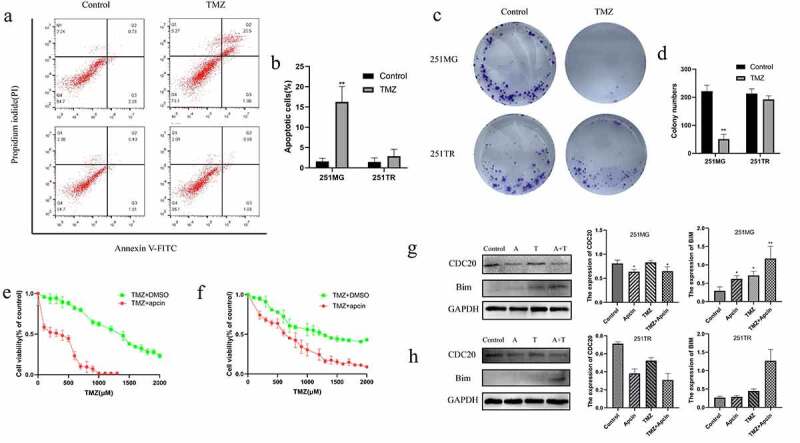
Apcin improves glioma sensitivity to temozolomide. (a, b) U251MG or U251TR cells were exposed to increase the concentrations of TMZ in culture medium for 48 h. Cell viability was measured by a PI/FITC-annexin V assay. (c, d) Colony formation assay showing U251TR cells exhibited lower sensitivity to TMZ than the U251MG cells. (e) U251MG cells were exposed to increase the concentrations of TMZ or TMZ+30 μM apcin in culture medium for 48 h. (f) U251TR cells were exposed to increase the concentrations of TMZ or TMZ+30 μM apcin in culture medium for 48 h. (g,h) Western blot analysis of bim in U251MG/U251TR cells and then treated with TMZ or apcin for 48 h (A: 30 μM apcin, T: 100 μM TMZ, A + T: treated with 30 μM apcin and 100 μM TMZ). Quantitative results of western blotting (*p < 0.05 vs. the control group, **p < 0.01 vs. the control group)

Subsequently, we further validated the relationship between apcin and glioma TMZ resistance. U251TR cells were treated with 200 μM TMZ for 48 h. We found that TMZ treatment significantly induced apoptosis in 251 MG cells but not 251TR cells (Figure 3(a,b)). The PI/FITC-annexin V assay showed that 251TR cells showed lower baseline concentration of apoptosis, indicating that the constructed U251 drug-resistant cell line was successful. U251TR cells exhibited lower sensitivity to TMZ than U251MG cells according to the colony forming assay (Figure 3(c,d)). After further addition of 30 μM apcin, U251MG and U251TR cells showed a significant increase in TMZ sensitivity (Figure 3(e,f)). In the control group, after adding equal amounts of dimethyl sulfoxide (DMSO), U251MG and U251TR cells still exhibited lower sensitivity to TMZ than the parental cells.

GBM cells were incubated in 30 μM apcin, 100 μM TMZ, or both for 48 h. The western blotting results showed that the expression of CDC20 was reduced and the expression of Bim was significantly increased in U251MG and U251TR cells after combined treatment with apcin and TMZ (Figure 3(g,h)). These results suggest that apcin targets inhibition of CDC20 expression enhances TMZ sensitivity in glioma cells.

## Discussion

GBM is the most malignant type of glioma, and TMZ is the primary treatment. Although many recent trials of targeted therapies have not shown significant efficacy, the discovery and application of precise biomarkers will increase the chances of treatment success. A deeper understanding of molecular biology will also guide an integrated therapeutic approach. Indeed, more molecular marker trials are still needed to improve our ability to evaluate these new targeted therapies [17]. However, most patients develop resistance to TMZ during treatment [18]. A comprehensive understanding of the molecular mechanisms involved in TMZ treatment resistance in glioma could help extend overall patient survival in individualized therapy.

Our previous study found that CDC20 expression increased with the malignant progression of glioma and led to poor patient prognosis [19]. In fact, it has been shown that the expression of CDC20 plays an important role in TMZ resistance in glioma cells [20]. CDC20 expression is upregulated in most tumors, which indicates that patients have poor prognosis. Therefore, CDC20 expression can be used as a biomarker for tumor prognosis and a therapeutic target for human cancers [21]. Therefore, inhibition of CDC20 expression may be an effective cancer treatment. Interfering with CDC20 expression can inhibit the growth and invasion of osteosarcoma cells [22], but the effect on glioma cells has not been well studied. Curcumin was shown to inhibit CDC20 expression in pancreatic cancer cells, and the discovery of specific CDC20 inhibitors for cancer treatment would be beneficial [23]. In addition, Wang et al. demonstrated that overexpression of CDC20 facilitated TMZ resistance in glioma cells by activating the epithelial-mesenchymal transition signaling pathway [20]. In particular, this study, compared with previous studies, will help provide a theoretical basis and guidance for molecularly targeted therapy for GBM in the future [24]. These findings further highlight the potential of CDC20 inhibition to effectively treat GBM.

Apcin was first discovered in mitotic *Xenopus* egg extracts and is an inhibitor of cell cycle proteins [25]. Apcin has been found to promote prostate cancer cell apoptosis [26], inhibits osteosarcoma cell growth, and suppresses embryo implantation [27]. Apcin may be an effective cancer treatment. The role of apcin in glioma cells is poorly understood. Gao et al. found that apcin effectively inhibited osteosarcoma cell growth at concentrations of 50–75 μM [14]. In our preliminary experiments, we found that almost all U251MG cells died after 1 h of exposure to 75 μM apcin. Therefore, we selected a concentration gradient of apcin in the range of 0–60 μM. Our results showed that apcin inhibited GBM cell growth in a dose- and time-dependent manner. Moreover, apcin effectively inhibited the migration of GBM cells. In addition, we found that apcin could effectively enhance the sensitivity of GBM cells to TMZ by using a CCK-8 assay.

Bim has been shown to act as an apoptotic activator. Bim overexpression inhibits tumor growth and drug resistance, and various chemotherapeutic agents regulate Bim expression [28]. CDC20 can inhibit apoptosis by targeting Bim for ubiquitination [5]. Bim can induce the release of cytochrome C into the cytoplasm, followed by PARP cleavage, leading to apoptosis [29]. We found elevated expression of Bim in glioma cells after the use of apcin, and the expression increased with higher doses of apcin. Gambichler et al. demonstrated a significant negative correlation between a poor prognosis and low Bim protein expression in melanoma patients and confirmed that Bim is an independent predictor of advanced melanoma [30]. Low expression of Bim after siRNA transfection delays paclitaxel-induced apoptosis, suggesting that the downregulation of Bim is responsible for tumor resistance to chemotherapeutic agents [31]. These studies suggest that overexpression of Bim enhances the efficacy of oncologic chemotherapy. The western blotting results showed that after the combined use of apcin and TMZ, the expression of Bim was significantly higher than that in the control group, the apcin-alone group and the TMZ-alone group. It was further shown that apcin can promote the apoptosis of GBM cells by inducing Bim expression, thereby increasing the sensitivity of GBM cells to TMZ. These results suggest that apcin exhibits antitumor activity in GBM cells; therefore, CDC20 is a promising molecular target, and apcin has great potential to guide the treatment of GBM and combat TMZ resistance in the future.

## Conclusion

The results show that apcin can effectively inhibit GBM growth and improve TMZ sensitivity. Apcin has the potential to treat GBM and is expected to provide new ideas for individualized treatment.
